# Hospital Gazettes and Journals

**Published:** 1920-10-09

**Authors:** 


### Hospital Gazettes and Journals.

*St. Bartholomew's Hospital Journal.*

We take this all-too-true paragraph from an article entitled "The First Year":—

"In three-quarters of a year you must have a thorough groundwork of three of the leading sciences of the day. You are rushed from the development of the nervous system in the chick to the laws of mass action in chemistry, and thence to the theory of light-waves in physics. You comfort yourself with the fact that the University only requires an introductory knowledge of these subjects, treated in an elementary manner. You carry out their instructions most implicitly, and feel that it's jolly to be alive and easy to be a medical student. You get into the 'smattering of everything' mood. You dance lightly through pages of heavy theory, with one eye fixed on the 'elementary knowledge' clause in the syllabus. You listen to your lecturer with tolerant pity. Poor fool! He has such a depth of knowledge, and has lectured for fifty years on the same subjects. But he has forgotten to read the *syllabus!*"

Sequel: The University in July passed less than 50 per cent. of all candidates for the first examination for medical degrees!

*Guy's Hospital Gazette.*

The *Gazette's* mysterious and remarkable Chinese correspondent, Ho Ho, writes this month on the subject of Guyoscopy. Referring to Guyos, on whose principles the science was founded, he says:—

"But all the experiences of his strange youth and the acquisitions of his remarkable adolescence were but preparatory to the realisation of his profound purpose in the promulgation of the principle of Guyoscopy. What, then, is Guyoscopy? This is the question I have come before you to answer. Guyoscopy is a science; yet it is more than a science. Guyoscopy is an art, yet it is greater than an art. It is, in a word, or rather two words, an artful science. It consists in doing as they do at Guy's, the home of Guyos.

"But if you ask me, as a question, what they do at Guy's, I must answer you after the manner of a great Chinese obstetrician who was asked 'How can a doctor be certain of pregnancy?' and his reply was, 'Wait and see.'"

We believe that most voluntary hospitals are at the present time, perforce, carrying out this advice.

